# Baicalein inhibits heparin-induced Tau aggregation by initializing non-toxic Tau oligomer formation

**DOI:** 10.1186/s12964-021-00704-3

**Published:** 2021-02-12

**Authors:** Shweta Kishor Sonawane, Vladimir N. Uversky, Subashchandrabose Chinnathambi

**Affiliations:** 1 Neurobiology Group, Division of Biochemical Sciences, CSIR-National Chemical Laboratory (CSIR-NCL), Dr. Homi Bhabha Road, Pune, 411008 India; 2 Department of Molecular Medicine and USF Health Byrd Alzheimer’s Research Institute, Morsani College of Medicine, University of South Florida, Tampa, FL 33612 USA; 3 Academy of Scientific and Innovative Research (AcSIR), Ghaziabad, 201002 India

**Keywords:** Baicalein, Tau inhibition, Tau oligomers, Tau dissolution, Alzheimer’s disease

## Abstract

**Background:**

Amyloid aggregate deposition is the key feature of Alzheimer’s disease. The proteinaceous aggregates found in the afflicted brain are the intra-neuronal neurofibrillary tangles formed by the microtubule-associated protein Tau and extracellular deposits, senile plaques, of amyloid beta (Aβ) peptide proteolytically derived from the amyloid precursor protein. Accumulation of these aggregates has manifestations in the later stages of the disease, such as memory loss and cognitive inabilities originating from the neuronal dysfunction, neurodegeneration, and brain atrophy. Treatment of this disease at the late stages is difficult, and many clinical trials have failed. Hence, the goal is to find means capable of preventing the aggregation of these intrinsically disordered proteins by inhibiting the early stages of their pathological transformations. Polyphenols are known to be neuroprotective agents with the noticeable potential against many neurodegenerative diseases, such as Alzheimer’s, Parkinson’s, and Prion diseases.

**Methods:**

We analyzed the capability of Baicalein to inhibit aggregation of human Tau protein by a multifactorial analysis that included several biophysical and biochemical techniques.

**Results:**

The potency of Baicalein, a polyphenol from the *Scutellaria baicalensis Georgi*, against in vitro Tau aggregation and PHF dissolution has been screened and validated. ThS fluorescence assay revealed the potent inhibitory activity of Baicalein, whereas ANS revealed its mechanism of Tau inhibition viz. by oligomer capture and dissociation. In addition, Baicalein dissolved the preformed mature fibrils of Tau thereby possessing a dual target action. Tau oligomers formed by Baicalein were non-toxic to neuronal cells, highlighting its role as a potent molecule to be screened against AD.

**Conclusion:**

In conclusion, Baicalein inhibits aggregation of hTau40 by enhancing the formation of SDS-stable oligomers and preventing fibril formation. Baicalein-induced oligomers do not affect the viability of the neuroblastoma cells. Therefore, Baicalein can be considered as a lead molecule against Tau pathology in AD.

**Video Abstract**

## Background

**Fig. 1:**
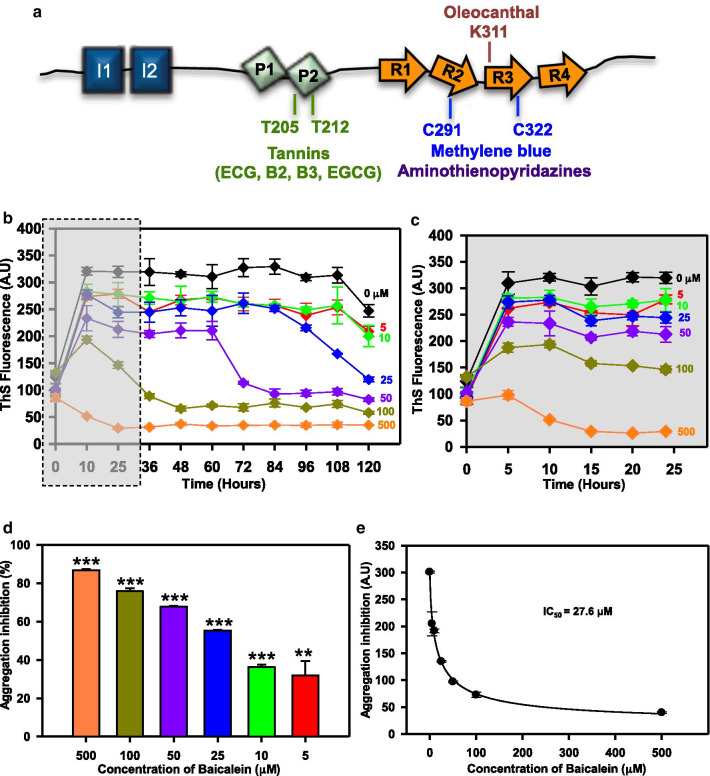
Tau aggregation inhibition by Baicalein. **a** Tau protein domain organization with the known binding sites for the small molecule inhibitors of Tau aggregation. **b** ThS fluorescence for aggregation kinetics of full-length Tau in presence of varying concentrations of Baicalein. The ThS fluorescence decreased with increase in dose of Baicalein showing concentration dependent inhibition of Tau aggregation. **c** ThS fluorescence for Baicalein treated samples at the initial time point revealed that the higher concentrations had rapid effect on inhibiting Tau aggregation within first 24 h. The ThS fluorescence intensity decreased rapidly in the 24 h time window and remained at basal level throughout the kinetics. **d** The ThS percent inhibition of Tau aggregation at different concentrations of Baicalein. The highest concentration of Baicalein 500 μM showed around 85% inhibition followed by 75% by 100 μM of Baicalein. **e** The IC_50_ value for full-length Tau aggregation inhibition, which was found to be 27.69 μM of Baicalein. (The values are mean ± std. deviation of two independent experiments. The statistical analysis was carried out by Student’s unpaired *t* test with respect to Baicalein untreated control. ***p ≤ 0.001, **p ≤ 0.01, *p ≤ 0.05. *ns* non-significant *p* value). Further, post hoc analysis was carried by one-way ANOVA and Tukey’s criterion was determined for honestly significant difference (HSD). The data was considered significant if |X–X′| > Tukey’s criterion

Alzheimer’s disease (AD) is the leading cause of dementia and cognitive inabilities in aged population [1, 2]. The disease results in a progressive neuronal dysfunction and neuronal atrophy followed by death [3]. Protein misfolding and aggregation within the neurons and on the neuronal membranes hamper cellular transport and synaptic transmission and cause neuronal dysfunction. Two functionally distinct and discrete proteins are involved in the neuronal dysfunction associated with AD. The intra-neuronal functional loss is caused by the neurofibrillary tangles of microtubule-associated protein Tau [4]. The proteinaceous deposits of Aβ peptide proteolytically derived from the amyloid precursor protein (APP) on the neuronal membrane affects its synaptic transmission [5]. Both pathological processes together cause neuronal death. The amyloid cascade hypothesis was widely accepted as the key pathological mechanism triggering AD until recent finding that the aberrant APP metabolism triggers the disease and not the Aβ plaques deposition. This reconsideration of Aβ hypothesis has also pointed out to the crucial role of Tau pathology as one of the key determinants of AD [6]. The reconsideration of Aβ as a trigger in AD was necessitated by the fact that the therapies against Aβ accumulation [7], though were effective in reducing the plaque load, were not able to improve the symptoms of AD [8]. Hence, the focus of AD therapeutics has shifted towards Tau as a target. Progression of AD can be described based on the Braak stages, which in turn mark the spread of Tau lesions and proportionate the symptoms of the disease [9, 10]. Being intrinsically disordered, Tau is a highly flexible protein, which compacts on temperature variations and adopts transient structures on interaction with its partners [11, 12]. Tau is modified post-translationally, which can either aid in its function or leads to its aggregation [13]. As a result of aberrant post-translational modifications (PTMs), Tau detaches from microtubules and self-assemble, resulting in the increased intracellular accumulation of aggregated forms [14]. The misfolded and aggregated Tau is also postulated to spread between neurons and aggravate the pathology [15]. The most extensive and important PTM involved in the pathological Tau aggregation is phosphorylation, and hence it is a greatly targeted modification to alleviate the disease [16]. Tau therapeutics can be categorized into wide array of molecules belonging to diverse classes that have specific interaction sites on Tau (Fig. 1a).

There is a repertoire of molecules that were found to be effective against Tau pathology. These compounds include cinnamaldehyde [17], olecanthal [18], azaphilones, resveratrol [19], curcumin [20], quercetin [21], polyphenols [22], liminoids [23], melatonin [24], Rose Bengal [25], etc [26, 27]. Natural polyphenolic compounds are emerging as neuroprotective molecules because of their ability to abrogate oxidative stress, which is one of the major causative agents of the age-related neurodegeneration [28, 29]. Polyphenols also have multitude of intracellular targets, which makes them efficient candidates for improving cognitive abilities and blocking neurodegeneration [30]. Though polyphenols are found to be effective against various molecular targets in neurodegenerative diseases, they have drawbacks that need to be addressed in depth [31]. Baicalein is the active flavone purified from the dried roots of *Scutellaria baicalensis Georgi*. This molecule has widespread functions in myriad of pathologies, such as cancer [32], oxidative stress [33], inflammation [34], bacterial [35] and viral infections [36], etc. Baicalein has been widely studied in neurodegenerative diseases and found to be potent against protein misfolding and aggregation [37]. Baicalein protects against the cerebral ischemia-mediated neuronal death, which is one of the major causes of death and disability world-wide [38]. Baicalein attenuates disease pathology in in vitro and in vivo models of Parkinson’s disease (PD). It inhibits aggregation of α-synuclein and abates inflammasome activation in the nigro-striatal dopaminergic system in vivo [39, 40]. The Prion protein-induced neurotoxicity, mitochondrial dysfunction and ROS production are suppressed by Baicalein in the SH-SY5Y and SK-N-SH cells [41]. Baicalein also plays a major role in overcoming AD pathologies. It improved Aβ-induced LTP impairments in mouse model via the phosphorylation of Akt, which is involved in cell survival and growth [42]. Baicalein prevented Tau hyperphosphorylation by inhibiting GSK-3β activity. This flavone also rescued the behavioral deficits in AD model induced by AlCl_3_ [43]. All these studies depicted the role of Baicalein in animal models of the neurodegenerative diseases at the physiological level. Our aim is to study the direct effect of Baicalein in inhibiting Tau aggregation and a putative mechanism for its action. Here, we report that Baicalein inhibits heparin-induced Tau aggregation by initializing Tau oligomer formation, dissolving these oligomers and preventing their further fibrillization.

## Experimental procedures

### Chemicals and reagents

Baicalein, ThS, ANS, BES, and MTT were purchased from Sigma. Other reagents such as NaCl, Sodium azide, heparin, MgCl_2_, EGTA and PMSF were obtained from MP Biomedicals. DTT was obtained from Calbiochem and protease inhibitor cocktail was purchased from Roche. pan-Tau K9JA was purchased from Dako (A-0024) and secondary goat anti-rabbit antibody conjugated to HRP was purchased from Thermo Fisher Scientific (A16110). Hybond-PVDF 10600087 membrane 0.45 μm was obtained from Amersham Biosciences. The neuroblastoma N2a (ATCC: CCL-131 Neuro-2a Neuroblastoma mouse) cells were purchased from ATCC. The cell culture reagents were purchased form Invitrogen. 400 carbon coated copper grids were purchased from Ted Pella (01822-F). 10 mM and 1 mM stock solutions of Baicalein were prepared in ethanol. 12.5 mM mother stock of ThS was prepared in 1:1 ethanol: miliQ water, which was further diluted to 200 μM stock in filtered miliQ water. ANS was prepared as 10 mM stock in filtered miliQ.

### Tau purification

The recombinant wild type human full-length Tau (hTau40WT) was expressed in *E. coli* BL21* cells by 0.5 mM IPTG induction at 37 °C for 4 h [11, 44–46]. The cells were harvested after induction and mechanically disrupted in a homogenizer (Constant Cell Disruption Systems) at 15,000-psi pressure. The cell lysate was supplemented with 0.5 M NaCl and 5 mM DTT and heated at 90 °C for 15 min. After cooling, the precipitated proteins were separated by centrifugation at 40,000 rpm for 50 min. The supernatant containing Tau was dialyzed overnight in Sepharose A buffer (20 mM MES, 1 mM EGTA, 1 mM MgCl_2_, 2 mM DTT, 0.1 mM PMSF pH 6.8). The dialysate was further cleared by centrifugation at 40,000 rpm for 50 min. Tau was further purified by cation-exchange column chromatography (Sepharose fast flow GE healthcare). The eluate obtained after cation-exchange chromatography was further purified by size-exclusion chromatography (16/600 Superdex 75 pg GE healthcare). The protein concentration was estimated by BCA assay. The protein was concentrated and stored in − 80 °C.

### Tau aggregation inhibition assay

Aggregation inhibition of full-length Tau in presence of Baicalein was performed as follows [27, 44]. Fresh aliquot of full-length Tau was taken from − 80 °C and centrifuged at 60,000 rpm for 1 h (Optima Max XP Beckman Coulter). The supernatant was separated and used for assay. The aggregation assay was set up with 20 μM of Tau in 20 mM BES buffer pH 7.4, protease inhibitor cocktail, 0.01% Sodium azide. 5 μM of Heparin (17,500 Da) was used as an inducer of aggregation. 25 mM NaCl and 1 mM DTT were added to the reaction mixture to provide ionic strength and reducing conditions respectively. Baicalein was added in the concentrations of 0–500 μM and the respective reaction mixtures were incubated at 37 °C.

### Soluble protein assay

In order to check the effect of Baicalein in absence of the aggregation inducer heparin, a soluble protein assay was set up. The assay composition was same as mentioned above. Baicalein 25 and 100 μM respectively were incubated with 20 μM Tau in presence and absence of inducer heparin in separate reaction mixtures. Two control reaction mixtures were set up for without Baicalein but presence and absence of heparin with 20 μM Tau. All four reaction mixtures were incubated at 37 °C.

### PHFs formation in vitro and Tau disaggregation assay

The mature fibrils for full-length Tau were prepared as follows. 100 μM soluble Tau was incubated with 25 μM Heparin 17,500 Da in the aforementioned reaction buffer. The mature fibril formation was allowed for 8 days at 37 °C. The disaggregation assay was set up by incubating 20 μM of mature fibrils with varied concentrations of Baicalein (0 to 500 µM) and the dissolution of fibrils was recorded by fluorescence, SDS and TEM analysis.

### ThS fluorescence assay

The progression of Tau aggregation was monitored by Thioflavin S fluorescence assay [23, 47]. 2 μM of Tau from the reaction mixture was incubated with 8 μM ThS in 50 mM ammonium acetate pH 7.0 for 10 min. The readings were recorded in triplicates by exciting the fluorophore at 440 nm and obtaining the emission readings at 521 nm in Tecan Infinite 200 Pro series plate reader. The buffer background fluorescence was subtracted from each reading. Initial measurements were taken within an interval of 5 h till 25 h and later readings were continued at an interval of 12 h each.

### ANS fluorescence assay

The hydrophobicity changes in Tau protein during the process of aggregation were monitored by ANS fluorescence measurement. 2 μM Tau was incubated with 40 μM ANS in 50 mM ammonium acetate pH 7.0 for 20 min. The fluorescence measurements were carried out at excitation/emission wavelengths of 390 and 475 nm respectively in Tecan Infinite 200 Pro series plate reader.

### SDS-PAGE analysis

SDS-PAGE analysis was carried out for the detection confirming Tau aggregation inhibition on 10% polyacrylamide gels at 3 time intervals (0, 48, and 120 h). The SDS-PAGE gels were quantified by Image Lab software (Bio-Rad). The quantification for complete individual lanes was carried out using the software and the obtained intensities were plotted in the form of the bar graphs.

### Immunoblotting

The samples at regular time intervals were confirmed for aggregation inhibition by immunoblotting using the antibody against total pan-Tau K9JA (Dako A-0024). The samples from 20 μM reaction mixtures were diluted 10 folds and loaded onto the SDS-PAGE for electrophoresis. The subsequent proteins were transferred onto Hybond-PVDF membrane 0.45 μm (Amersham Biosciences) at 200 mA for 90 min. After the transfer, the blot was blocked in 5% milk in PBST (0.1% Tween 20) for 1 h at room temperature. Subsequently, the blot was incubated with primary antibody rabbit polyclonal anti-human Tau K9JA diluted 1:8000 for 1 h at room temperature. The unbound antibody was removed by washing the blot in PBST (0.1%Tween 20) thrice. The blot was further probed with secondary goat anti-rabbit antibody conjugated to HRP for one hour at room temperature in 1:10,000 dilutions. The unbound antibody was washed thrice with PBST for 10 min each and the blot was developed using ECL-Plus reagent (Thermo Fisher Scientific). The chemiluminescence was detected on Amersham Imager 600.

### Circular dichroism spectroscopy

The changes in Tau conformation during the process of aggregation in presence and absence of Baicalein were monitored by CD spectroscopy. The measurements were carried out in quartz cuvette (1 mm) in Jasco J-815 CD spectrometer. The scan was carried out at a speed of 100 nm/min in the range of 190–250 nm at 1 nm bandwidth. A total of 5 acquisitions were obtained for each sample. The samples were diluted to 3 µM in sodium phosphate buffer pH 6.8 for CD measurements.

### Electron microscopy measurements

Tau filaments in absence and presence of Baicalein were observed by transmission electron microscope Tecnai G2 20 S-Twin. 400-mesh carbon coated copper grids (Ted Pella 01822-F) were spotted with 2 μM Tau. The excess was blotted on a Whatman paper. Two quick washes of filtered MilliQ water were given for 45 s each and the grids were stained in 2% uranyl acetate for 1 min. The grids were properly dried before measurement.

### Cell viability assays

The neuroblastoma N2a (ATCC: CCL-131 Neuro-2a Neuroblastoma mouse) cells were used for the toxicity assays. 10,000 cells/well were seeded in 96 well culture plates and grown in complete DMEM F12 media supplemented with antibiotic Penstrep [48] for 24 h. The cells were treated with Baicalein (0 to 20 μM) with 5 μM full-length soluble Tau or 5 μl of Tau aggregates (50 μM total concentration) in serum-starved media (0.5% FBS) for 24 h. 0.5 mg/ml MTT (Methylthiazolyldiphenyl-tetrazolium bromide) was added to each well and incubated for four hours at 37 °C. The viable cells enzymes reduce MTT into formazan crystals which were dissolved in 100 µl of 100% DMSO. The reading was taken at 570 nm in Tecan Infinite 200 Pro series plate reader. The viability of untreated cells was considered as 100% and the viability of treatment groups was calculated in accordance with this untreated group.

### Statistical analysis

The error bars represent mean ± SD values. 95% confidence intervals. The statistical analyses were carried out by Sigma Plot 10.0. Unpaired *t* test was used to calculate the *p* values. *p value ≤ 0.05, **p value ≤ 0.01, ***p value ≤ 0.001. Further, post hoc analysis was carried by one-way ANOVA and Tukey’s criterion was determined for honestly significant difference (HSD). The data was considered significant if |X–X′|> Tukey’s criterion.

## Results

### Baicalein inhibits hTau40 assembly

Screening of Tau aggregation inhibitors (TAI) in cell-free assays represents a well-established approach. These primary screens include ThS fluorescence to study the progress of aggregation in solution. We adopted a similar preliminary screening approach to check the inhibitory effects of Baicalein (0–500 μM) on full-length Tau aggregation (Fig. 1b). We monitored ThS fluorescence initially at every 5 h till 24 h followed by the interval of 12 h. The initial 5 h interval measurements helped capturing the inhibition by higher concentrations of Baicalein (100, 500 μM). The fluorescence increased till 5 h for the higher concentrations followed by gradual decrease (Fig. 1c). This pattern of changes in fluorescence intensity was observed at lower Baicalein concentrations as well, but at increased incubation time. The fluorescence intensity did not decrease for the untreated Tau showing steady aggregation. This decrease in ThS fluorescence intensity in Baicalein-treated samples suggested concentration-dependent inhibition of Tau aggregation. The kinetics was monitored till 120 h and the percent inhibition at the end of 120 h was maximum 85% at 500 μM of Baicalein and 75% at 100 μM Baicalein (Fig. 1d). The IC_50_ value for inhibition of aggregation for full-length Tau was found to be 27.6 μM of Baicalein (Fig. 1e). Therefore, Baicalein was found to inhibit the assembly of full-length Tau aggregates at extended incubation time of 120 h.

### Baicalein is involved in the formation of Tau oligomers

**Fig. 2:**
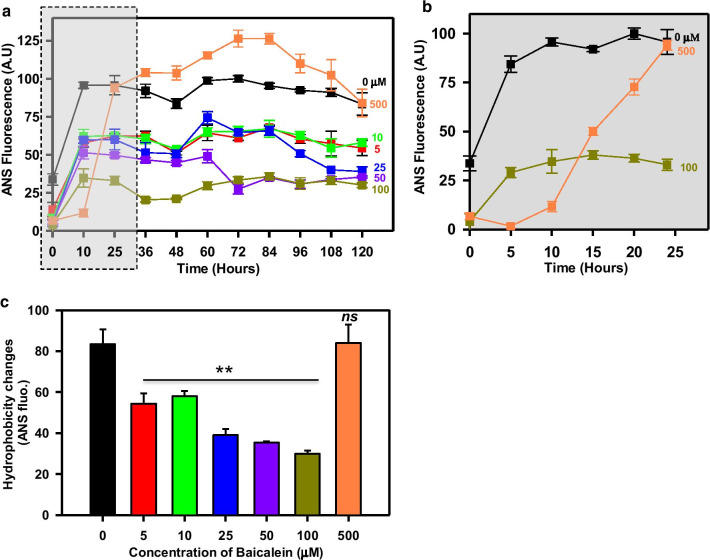
Tau oligomerization-induced by Baicalein. **a** ANS fluorescence for aggregation kinetics of full-length Tau in presence of varying concentrations of Baicalein. **b** The highest concentration (500 μM) of Baicalein induced Tau oligomerization within 15 h of incubation as evidenced by sharp rise in ANS fluorescence. **c** The hydrophobicity changes at the end of 120 h revealed that 500 μM of Baicalein showed increased hydrophobicity which is characteristic property of oligomers. (The statistical analysis was carried out by Student’s unpaired *t* test with respect to Baicalein untreated control. ***p ≤ 0.001, **p ≤ 0.01, *p ≤ 0.05. *ns* non-significant *p* value). Further, post hoc analysis was carried by one-way ANOVA and Tukey’s criterion was determined for honestly significant difference (HSD). The data was considered significant if |X–X′|> Tukey’s criterion

The ThS fluorescence studies revealed a distinct pattern of initial surge followed by gradual decrease, which led us to study the transition species formed in presence of Baicalein by ANS fluorescence. ANS binds to intermediate partially folded states with transient secondary structure with less compactness. ANS fluorescence showed a similar pattern like ThS with an initial increase and a further decrease in fluorescence (Fig. 2a, b). The hydrophobicity changes mapped by ANS showed that 500 μM of Baicalein increased Tau hydrophobicity as compared to lower concentrations (Fig. 2c). But at the higher concentration of Baicalein, ANS showed rapid and substantial increase in fluorescence at the initial time points and then the decrease before becoming stagnant (Fig. 2a). The kinetics of ANS suggests that Baicalein might be involved in initial Tau oligomer formation restricting further fibrillization.

### SDS-PAGE and Immunoblot analysis confirms Tau oligomerization in the presence of Baicalein

**Fig. 3:**
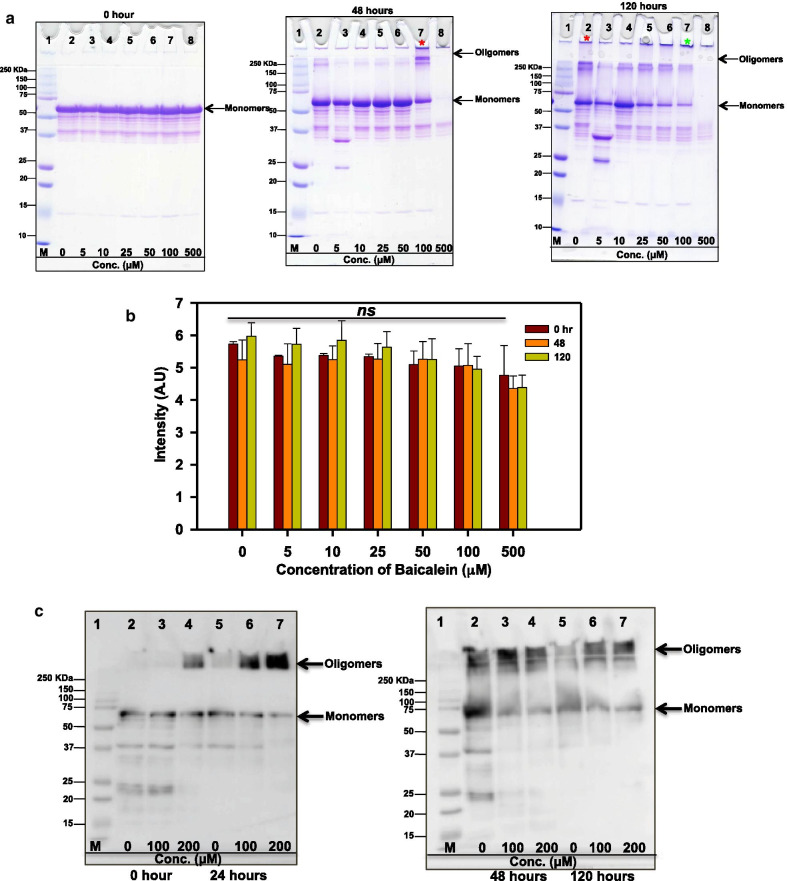
SDS-resistant Tau oligomers-induced by Baicalein. **a** SDS-PAGE analysis of Tau oligomers. The zero time point did not show presence of any aggregates in control as well as treated samples. At the end of 48 h higher order aggregates (red asterisk) were observed in the 100 μM Baicalein treated sample as compared to control. The 120 h incubated sample showed a decrease in aggregates (green asterisk) in treated samples as compared to control. **b** The SDS-PAGE quantification for different time points indicate the initial increase followed by the decrease in the aggregates at higher concentrations of Baicalein, which shows significant decrease in 500 µM Baicalein at 48 and 120 h. **c** The formation of Tau oligomers induced by Baicalein in presence of heparin was also confirmed by immunoblot analysis using the pan-Tau antibody K9JA for total Tau. At 24 h of incubation higher order oligomers were observed in Baicalein treated samples (lane 6, 7) as opposed to control (lane 2). After 48 h of incubation the oligomers population was seen to increase (lane 3, 4) but at 120 h the decrease in the oligomer load was observed (lane 7). (The statistical analysis was carried out by Student’s unpaired *t* test with respect to Baicalein untreated control. ***p ≤ 0.001, **p ≤ 0.01, *p ≤ 0.05. *ns* non-significant *p* value). Further, post hoc analysis was carried by one-way ANOVA and Tukey’s criterion was determined for honestly significant difference (HSD). The data was considered significant if |X–X′|> Tukey’s criterion

The fluorescence assays suggested role of Baicalein in inducing Tau oligomers. In order to reaffirm this hypothesis, reaction mixtures were analyzed at regular time intervals using the 10% SDS-PAGE. After 48 h of incubation, we observed a clear increase in higher molecular weight oligomers in sample treated with 100 μM Baicalein (48 h red asterisk) as compared to the control (Fig. 3a, b). At the same time point, ThS fluorescence decreased and ANS fluorescence began to increase suggesting the arrest of Tau oligomers by Baicalein. But the Tau sample treated with 500 μM Baicalein showed complete absence of higher order aggregates at 48 h suggesting complete inhibition of Tau assembly. As the time advanced, at the end of 120 h of incubation, we observed a clear decrease in the higher order aggregates in the 100 μM (120 h green asterisk) treated sample as compared to control (Fig. 3a, b). To confirm the aggregation inhibition of full-length Tau by Baicalein via promotion of oligomerization, we further carried out immunoblotting for these higher order aggregates by probing them with the total pan-Tau antibody (K9JA) at various time intervals. At zero time point, only soluble Tau protein could be visualized (Fig. 3c). The 24 h incubation showed complete oligomerization in the Tau samples treated with 200 μM Baicalein (all the material was present in the stacking gel part of the blot) as opposed to control, which showed mixed population of oligomers in the form of a smear in the resolving as well as stacking gel part of blot. The ANS fluorescence revealed that the treated samples showed increased oligomerization as the time of incubation progressed.

### Baicalein arrests Tau oligomers with partial secondary structure

**Fig. 4:**
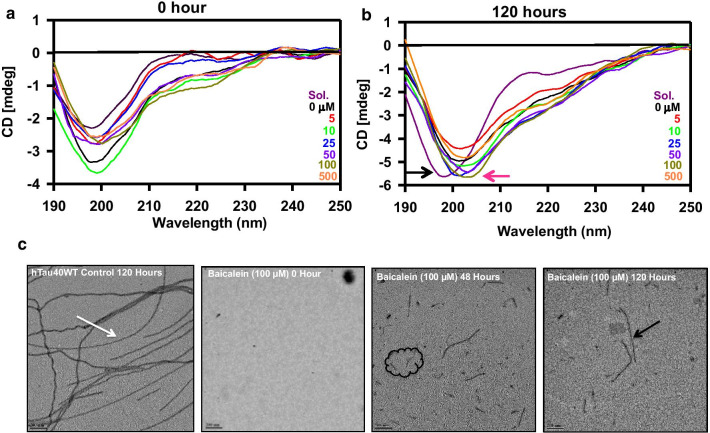
Tau aggregation inhibition studied by CD spectroscopy. **a** The conformational analysis of Baicalein treated and control samples showed the typical spectra of a random coil structure. The Sol. represents soluble protein without heparin. **b** At the end of 120 h the pattern of spectrum remained same with respect to soluble (black arrow) and treated samples (pink arrow) showed a partial β-sheet conformation. **c** The control untreated sample shows the presence of long filaments (white arrows) at day 5. The treatment with 100 μM Baicalein at the initial time points showed presence of intermediately long filaments (white arrow) and small broken filaments. As the time advanced, Baicalein was shown to inhibit Tau aggregation as only broken pieces (highlighted by black border and black arrow) of Tau filaments were visualized in the electron micrographs

For many aggregating intrinsically disordered proteins, oligomers are partially folded intermediate species formed during the process of aggregation. In order to check for the conformational states of Baicalein-induced oligomers, far-UV circular dichroism (CD) spectroscopic analysis was performed. Tau is a natively unfolded (intrinsically disordered) protein with a characteristic far-UV CD spectrum possessing prominent minimum at 198 nm and not showing significant signal at 220 nm (Fig. 4a). On the other hand, the far-UV CD spectrum of Tau protein in the presence of Baicalein is consistent with the presence of partial β-sheet structure, suggesting that Baicalein was able to induce some partial folding of Tau protein, likely leading to the formation of oligomeric species (Fig. 4b). Next, these Baicalein-induced (or Baicalein-stabilized) oligomers were visualized by transmission electron microscopy (Fig. 4c). The untreated Tau showed long filamentous aggregates at 120 h. Baicalein-treated Tau showed small filaments at 48 and 120 h with lack of intact fibrillar aggregates (Fig. 4c). Therefore, these qualitative analyses along with the conformational studies suggested that Baicalein arrests Tau aggregation at the stage of oligomer formation and prevents complete fibrillation of this protein.

### Baicalein does not alter Tau conformation

**Fig. 5:**
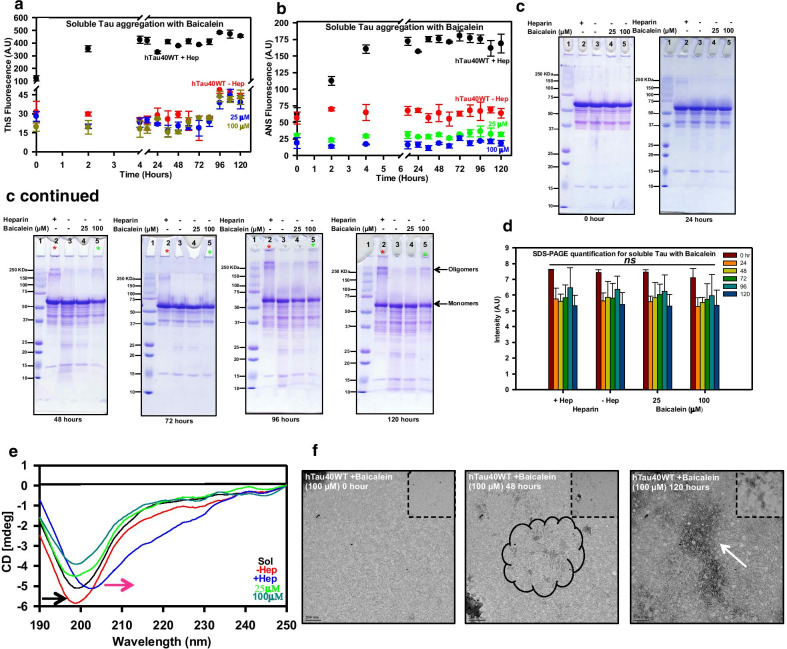
Interaction of Baicalein with soluble Tau. **a** The aggregation of Tau was monitored by ThS fluorescence. The positive control (heparin) showed an increase in intensity with respect to time as opposed to heparin negative control. The Baicalein treated soluble protein did not show any increase in fluorescence as compared to positive control suggesting that Baicalein does not lead to Tau fibrillization in absence of heparin. **b** The hydrophobicity imparted by Baicalein alone without heparin was monitored by ANS fluorescence. The intensity did not increase significantly as compared to the positive control. **c** The SDS-PAGE analysis revealed a time dependent increase in higher molecular aggregates in the positive control from 24 h of incubation (lane 2). No higher molecular weight aggregates were observed in negative control as well as sample treated with 25 μM of Baicalein (lanes 3, 4 respectively) but faint bands of higher order oligomers were observed in 100 μM treated sample from 72 h onwards (lane 5), suggesting formation of oligomers. **d** Quantification for SDS-PAGE for soluble Tau with Baicalein. **e** Conformational analysis of soluble Tau with Baicalein. The CD analysis showed normal spectra for negative control and Baicalein treated soluble Tau but a slight shift in the positive control (aggregated) Tau. **f** The electron micrographs at different time points reveal presence of amorphous aggregates at the initial time points and clumps of oligomers at the end point. (The statistical analysis was carried out by Student’s unpaired *t* test with respect to Baicalein untreated control. ***p ≤ 0.001, **p ≤ 0.01, *p ≤ 0.05. *ns* non-significant *p* value). Further, post hoc analysis was carried by one-way ANOVA and Tukey’s criterion was determined for honestly significant difference (HSD). The data was considered significant if |X–X′|> Tukey’s criterion

All the biochemical and biophysical experiments suggested induction (or stabilization) of Tau oligomers by Baicalein in the presence of heparin. In order to check whether Baicalein in the absence of heparin can cause conformational changes in Tau, this protein was incubated with Baicalein in absence of heparin. The ThS and ANS fluorescence did not increase for Baicalein-treated samples (in absence of heparin) (Fig. 5a, b). The SDS-PAGE analysis showed the presence of higher order structures in the heparin treated Tau. The sample treated with higher concentration of Baicalein (100 μM) showed presence of very faint higher order bands at a later time point 120 h (Fig. 5c, d). The fluorescence kinetics and the SDS-PAGE analysis clearly suggest that Baicalein might be able to induce Tau oligomerization but at a much slower pace. The structural analysis of these oligomers revealed that in the absence of heparin, Baicalein does not induce transition of Tau to β-sheet structure and maintains the native random coil conformation (Fig. 5e). The qualitative electron microscopy analysis showed that Baicalein-treated Tau without heparin does not form fibrils (Fig. 5f).

### Baicalein dissolves the mature fibrils of the aggregated Tau protein

**Fig. 6:**
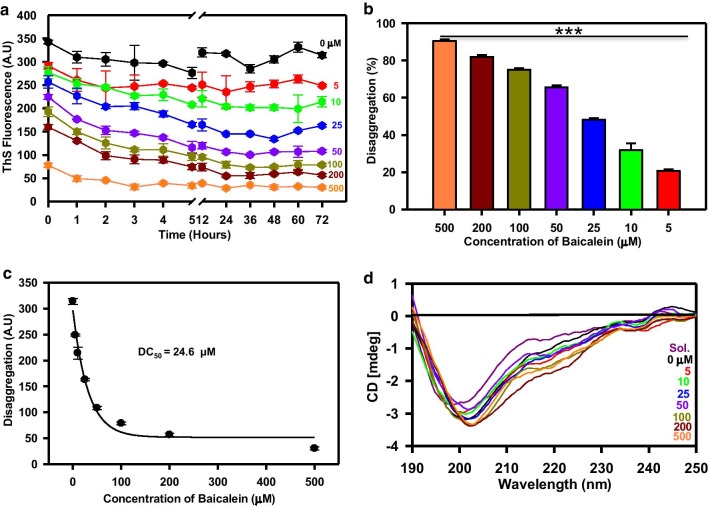
Dissolution of Tau aggregates by Baicalein. **a** The effect of Baicalein on the dissolution of mature Tau aggregates was studied using ThS fluorescence, which showed a decrease after 5 h of incubation in a concentration-dependent manner. **b** The percent disaggregation plot shows a maximum of 85% disaggregation by 500 μM of Baicalein treatment. **c** The plot shows the DC50 value for Tau disaggregation as 24.6 μM. **d** The CD analysis of disaggregated Tau by Baicalein shows the presence of mixed structures. The Sol. represents for soluble protein. (The statistical analysis was carried out by Student’s unpaired *t* test with respect to Baicalein untreated control. ***p ≤ 0.001, **p ≤ 0.01, *p ≤ 0.05. *ns* non-significant *p* value). Further, post hoc analysis was carried by one-way ANOVA and Tukey’s criterion was determined for honestly significant difference (HSD). The data was considered significant if |X–X′|> Tukey’s criterion

**Fig. 7:**
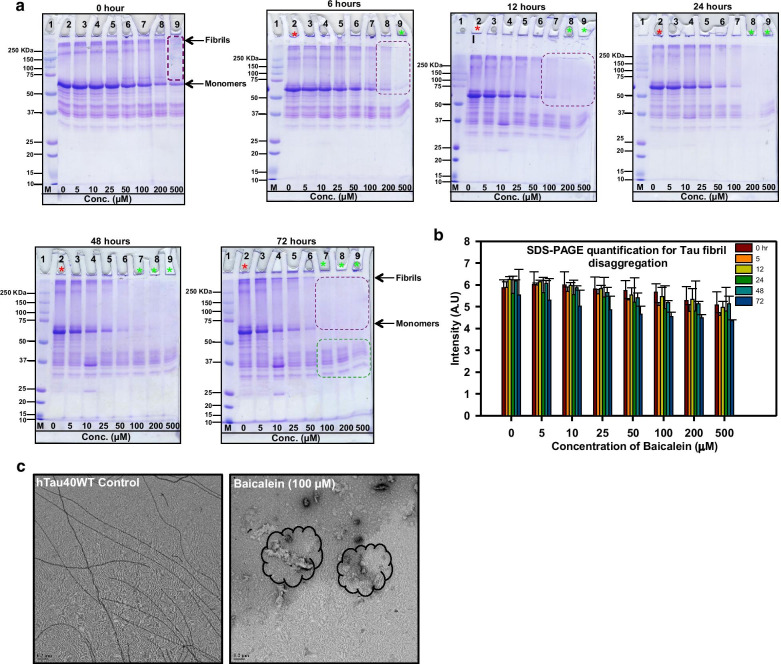
Baicalein-mediated Tau dissolution by SDS-PAGE. **a** Figure shows the time-dependent SDS-PAGE analysis of Tau aggregates dissolution. After 72 h, the higher concentrations of Baicalein showed a maximum decrease in the aggregates (lane 7, 8, 9 green asterisk) as compared to control (lane 2 red asterisk). **b** The SDS-PAGE quantification for different time points indicate the initial increase followed by the decrease in the aggregates at the higher concentrations of Baicalein. **c** Transmission electron micrographs showed the untreated mature fibrils (white arrow) versus disaggregated amorphous (encircled black), Baicalein treated fibrils of full-length Tau. (The statistical analysis was carried out by Student’s unpaired *t* test with respect to Baicalein untreated control. ***p ≤ 0.001, **p ≤ 0.01, *p ≤ 0.05. *ns* non-significant *p* value). Further, post hoc analysis was carried by one-way ANOVA and Tukey’s criterion was determined for honestly significant difference (HSD). The data was considered significant if |X–X′|> Tukey’s criterion

Although, Tau oligomers act as a toxic species, the filamentous Tau increase the aggregate load in the neuron, which hampers the cellular functioning, as well as increases the load on the clearance machinery [49]. Hence, it is important to have some efficient means that could dissolve these filamentous Tau aggregates to non-harmful oligomers. Since Baicalein had the inhibitory effect on Tau aggregation, we were interested to see whether this flavone can dissolve the pre-formed mature fibrils. To this end, the mature fibrils of full-length Tau were treated with series of concentration of Baicalein (0–500 μM). ThS fluorescence was used to monitor the disaggregation. The fluorescence intensity was found to decrease in the Baicalein-treated samples (Fig. 6a), with disaggregation approaching 90% in the presence of 500 μM Baicalein whereas the fluorescence of untreated control remained almost stagnant (Fig. 6b). The DC_50_ value for the disaggregation of fibrils formed by the full-length Tau was found to be 24.6 μM (Fig. 6c). The conformational analysis of disaggregated samples suggested the presence of mixed population of Tau and its aggregates (Fig. 6d). The SDS-PAGE analysis revealed a time- and concentration-dependent decrease in the aggregate load in the Baicalein-treated samples (Fig. 7a, b). The electron micrographs showed the presence of fibrillar aggregates in the control, but only amorphous aggregates and oligomers in the Baicalein-treated samples (Fig. 7c). These observations suggest that Baicalein has a dual role, being able to inhibit Tau fibrillation and to disaggregate the preformed Tau fibrils.

### Baicalein is biocompatible and rescues Tau aggregate-mediated toxicity in neuronal cells

**Fig. 8:**
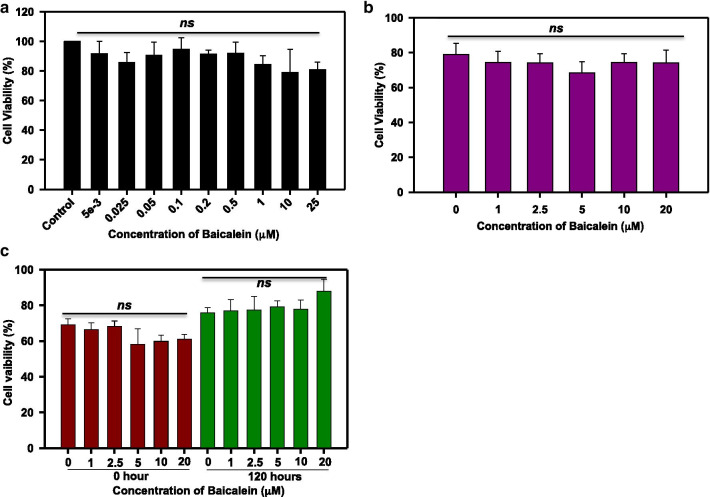
Cell toxicity of Baicalein. **a** N2a cells treated with a broad range of Baicalein shows no toxicity till 25 μM. **b** Baicalein treated soluble Tau shows no significant effect on cell viability implying Baicalein-induced oligomers are non-toxic. **c** Baicalein treated Tau aggregates shows a mild rescue of toxicity at 120 h with the highest concentration of Baicalein suggesting that disaggregated Tau mature filaments are non-toxic to neuro2a cells. The viability of untreated cells was considered as 100% and the viability of treatment groups was calculated in accordance with this untreated group. (The statistical analysis was carried out by Student’s unpaired *t* test with respect to Baicalein untreated control. ***p ≤ 0.001, **p ≤ 0.01, *p ≤ 0.05. ns: non-significant *p* value). Further, post hoc analysis was carried by one-way ANOVA and Tukey’s criterion was determined for honestly significant difference (HSD). The data was considered significant if |X–X′|> Tukey’s criterion

To assess the toxicity of Baicalein to neuronal cells, we screened a broad range of Baicalein concentrations from 5 nM to 20 μM for toxicity to neuroblastoma (N2a) cells. Baicalein was found to be non-toxic even at 25 μM concentration (Fig. 8a). To demonstrate the toxicity of Baicalein-treated Tau, cells were exposed to the Tau aggregation reaction mixtures containing various concentrations of Baicalein. Tau:Baicalein ratios were maintained as (1:0.25, 1:0.5, 1:1, 1:2 and 1:4) for 120 h. Baicalein-treated full-length Tau was found to be non-toxic to the neuronal cells (Fig. 8b), and the morphology of treated cells remained unchanged. As Baicalein disaggregated preformed Tau fibrils, we tested the effect of such disaggregated Tau on the N2a cells. Tau aggregates were treated with various concentrations of Baicalein and given to cells at 0 and 120 h. The highest concentration of Baicalein was shown to rescue the toxicity of Tau aggregates at 120 h and the cell morphology remained intact (Fig. 8c). Therefore, Baicalein-induced oligomers as well as Baicalein-treated mature fibrils are non-toxic to N2a cells.

## Discussion

**Fig. 9:**
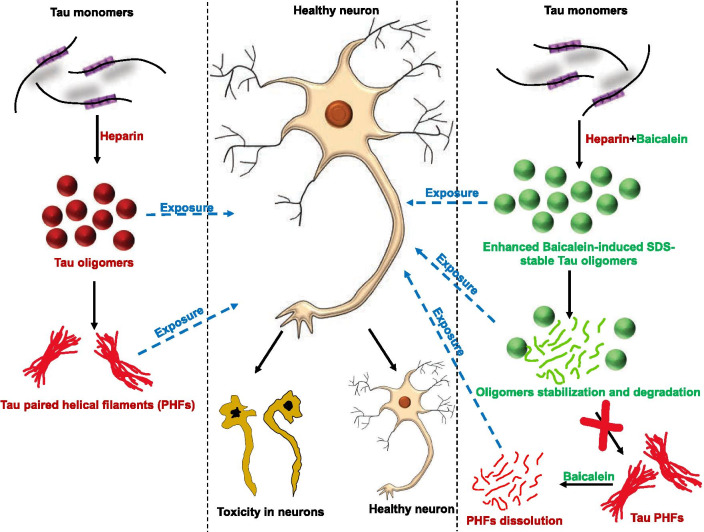
Dual effect of Baicalein on Tau. Tau in presence of heparin forms mature fibrils via intermediate oligomers formation. On the other hand, Tau in presence of Baicalein and heparin is restricted to form Tau oligomers without further mature fibril formation. These PHFs when exposed with Baicalein are dissolved into smaller fragments. The exposure of heparin-induced Tau oligomers and PHFs to healthy neuronal cells imparts toxicity in neurons and leads to neuronal death. The Baicalein-induced Tau oligomers as well as the dissolved fragments of PHFs do not affect the neuronal health and maintains the morphology. These suggest nontoxic nature of Baicalein-induced Tau oligomers

Tau as a target in AD has been extensively studied, and a great number of molecules were found to be potent against Tau-mediated pathologies. Baicalein has been implicated in overcoming AD pathologies with the major targets being ROS generation and rescuing of the behavioral and cognitive deficits [42]. The behavioral and cognitive deficits are late manifestations of Tau pathology, which starts with abrupt aggregation followed by gradual accumulation of aggregated material. In the current scenario, the pathological transitions of Tau at the initial stages need to be inhibited. Few molecules, including methylene blue, azaphilones, anthraquinones, etc. have been found to inhibit Tau aggregation at the initial stages. Many molecules like cinnamaldehyde [50], phenothiazines [51], aminothienopyridazines [52] and vitamin B12 [53] screened against Tau aggregation are known to modify the cysteine residues which are essential in mediating Tau aggregation [54]. Baicalein, a natural small molecule was shown to be inhibit the aggregation of α-synuclein, a key protein involved in the PD pathogenesis [55]. We report analogous results for human Tau protein, wherein Baicalein efficiently inhibits the protein aggregation in vitro (Fig. 9). Though, the IC_50_ of Baicalein for Tau inhibition is reported to be 2.7 μM [56], no sufficient evidence is available for the same. We report an IC_50_ of 27.8 μM for inhibition of Tau and DC_50_ of 24.6 μM. Similar for α-synuclein, Baicalein enhanced Tau oligomerization. In the presence of Baicalein, the aggregation process of Tau is halted at the stage of the oligomer formation, without allowing protein to form fibrils. This is evident from ANS fluorescence, which increases initially and then decreases at the extended incubation time. ANS fluorescence increases due to the binding of fluorophore to the exposed hydrophobic regions of Tau during aggregation [57]. Thus, Baicalein enhances Tau oligomerization and captures these intermediate species. The structural studies revealed that the Baicalein-stabilized α-synuclein oligomers have a β-sheet-rich structure [58], which is in agreement with our data, wherein the conformational analyses of Baicalein-induced Tau oligomers revealed the presence of the partial β-sheet structure. Baicalein-induced Tau oligomers were found to be SDS-resistant. The SDS-stable Aβ oligomers are formed by green tea polyphenol EGCG via polar and non-polar interactions [59, 60]. Several other polyphenols, such as nordihydroguaiaretic acid (NDGA), resveratrol, and myricetin divert the Aβ monomers and fibrils towards SDS-stable oligomers [61]. The catecholamine dopamine inhibits α-synuclein fibrillization, as well as forms covalent adducts with this protein resulting in the formation of the SDS- and heat-stable oligomers [62]. The oligomers induced by these compounds are off the fibrillation pathway and non-toxic, which is similar to Baicalein-induced hTau40 oligomers characterized in our study. Though Baicalein is found to be potent in inhibiting aggregation of full-length Tau by inducing off-pathway oligomers, the autoproteolysis of Tau leading to its fragmentation may interfere with the observation. This inherent nature of Tau protein to fragment at higher incubation temperatures [63] still remains a major limitation in various in vitro studies of full-length Tau. Baicalein treatment inhibited the mature fibril formation for Tau, which was visualized by TEM. The treatment with bioflavonoid cinnamaldehyde show presence of broken Tau filaments and absence of intact fibrils [17]. Thus, bioflavonoids might have similar mechanisms for inhibiting the amyloidogenic aggregation of proteins. Baicalein disaggregated the pre-formed fibrils of Tau similar to α-synuclein fibril dissolution [55]. This shows that Baicalein might have a common pathway of inhibiting misfolded protein aggregation and dissolution of mature fibrils. Baicalein interacts with proteins in a residue-specific manner. For example, in human serum albumin it interacts with Leu, Arg, and Ala residues [64]. On the other hand, Baicalein binds close to the tyrosine residue of Aβ and forms Schiff’s base with the lysine residue [65]. Furthermore, the Baicalein-induced Tau oligomers were found to be non-toxic to the neuronal cells. Similarly, Baicalein rescued the toxicity of α-Synuclein oligomers [66]. Further, it also protected SH SY-5Y and HeLa cells from toxicity of α-Synuclein and Aβ oligomers thus proving to be a potential molecule against amyloid aggregation in general. The potency of Baicalein to be a therapeutic is hampered by its poor water solubility and low bioavailability. Nonetheless, various strategies are being applied to overcome this including cocrystal synthesis, biocompatible nano emulsions and microemulsions, which enhance the bioavailability of Baicalein [67, 68].

## Conclusion

In conclusion, our study suggests the potency of Baicalein in having a dual target action against two pathological Tau process. Baicalein efficiently inhibits Tau assembly by promoting off pathway oligomers as well as dissolves Tau PHFs. This highlights its potential in ameliorating multifactorial disease pathologies. Thus, Baicalein can act as an dynamic candidate for AD therapeutics (Fig. 9).

## Data Availability

All data generated or analyzed during this study are included in this manuscript.

## Abbreviations

IDPs: Intrinsically disorder proteins
PHFs: Paired helical filaments
ThS: Thioflavin S
ANS: 8-Anilinonaphthalene-1-sulfonic acid
CD: Circular dichroism
TEM: Transmission election microscopy
MTT: Methylthiazolyldiphenyl-tetrazolium bromide
